# The Effects of Oral Contraceptives on Exercise Performance in Women: A Systematic Review and Meta-analysis

**DOI:** 10.1007/s40279-020-01317-5

**Published:** 2020-07-14

**Authors:** Kirsty J. Elliott-Sale, Kelly L. McNulty, Paul Ansdell, Stuart Goodall, Kirsty M. Hicks, Kevin Thomas, Paul A. Swinton, Eimear Dolan

**Affiliations:** 1 Department of Sport Science, Sport Health and Performance Enhancement (SHAPE) Research Centre, Nottingham Trent University, Nottingham, UK; 2 Department of Sport, Exercise and Rehabilitation, Faculty of Health and Life Sciences, Northumbria University, Newcastle-upon-Tyne, UK; 3 School of Health Sciences, Robert Gordon University, Aberdeen, UK; 4 Applied Physiology and Nutrition Research Group, Escola de Educação Física e Esporte, Faculdade de Medicina FMUSP, Universidade de São Paulo, São Paulo, Brazil

## Abstract

**Background:**

Oral contraceptive pills (OCPs) are double agents, which downregulate endogenous concentrations of oestradiol and progesterone whilst simultaneously providing daily supplementation of exogenous oestrogen and progestin during the OCP-taking days. This altered hormonal milieu differs significantly from that of eumenorrheic women and might impact exercise performance, due to changes in ovarian hormone-mediated physiological processes.

**Objective:**

To explore the effects of OCPs on exercise performance in women and to provide evidence-based performance recommendations to users.

**Methods:**

This review complied with the Preferred Reporting Items for Systematic Reviews and Meta-Analyses guidelines. A between-group analysis was performed, wherein performance of OCP users was compared with naturally menstruating women, and a within-group analysis was conducted, wherein performance during OCP consumption was compared with OCP withdrawal. For the between-group analysis, women were phase matched in two ways: (1) OCP withdrawal versus the early follicular phase of the menstrual cycle and (2) OCP consumption versus all phases of the menstrual cycle except for the early follicular phase. Study quality was assessed using a modified Downs and Black Checklist and a strategy based on the recommendations of the Grading of Recommendations Assessment Development and Evaluation working group. All meta-analyses were conducted within a Bayesian framework to facilitate probabilistic interpretations.

**Results:**

42 studies and 590 participants were included. Most studies (83%) were graded as moderate, low or very low quality, with 17% achieving high quality. For the between-group meta-analysis comparing OCP users with naturally menstruating women, posterior estimates of the pooled effect were used to calculate the probability of at least a small effect (*d* ≥ 0.2). Across the two between-group comparison methods, the probability of a small effect on performance favouring habitual OCP users was effectually zero (*p* < 0.001). In contrast, the probability of a small effect on performance favouring naturally menstruating women was moderate under comparison method (1) (*d* ≥ 0.2; *p* = 0.40) and small under comparison method (2) (*d* ≥ 0.2; *p* = 0.19). Relatively large between-study variance was identified for both between-group comparisons ($\tau$_0.5_ = 0.16 [95% credible interval (CrI) 0.01–0.44] and $\tau$_0.5_ = 0.22 [95% CrI 0.06–0.45]). For the within-group analysis comparing OCP consumption with withdrawal, posterior estimates of the pooled effect size identified almost zero probability of a small effect on performance in either direction (*d* ≥ 0.2; *p* ≤ 0.001).

**Conclusions:**

OCP use might result in slightly inferior exercise performance on average when compared to naturally menstruating women, although any group-level effect is most likely to be trivial. Practically, as effects tended to be trivial and variable across studies, the current evidence does not warrant general guidance on OCP use compared with non-use. Therefore, when exercise performance is a priority, an individualised approach might be more appropriate. The analysis also indicated that exercise performance was consistent across the OCP cycle.

**Electronic supplementary material:**

The online version of this article (10.1007/s40279-020-01317-5) contains supplementary material, which is available to authorized users.

## Key Points

**Table:** 

When compared with a natural menstrual cycle, oral contraceptive pill (OCP) use might result in slightly inferior exercise performance, although any group level effect is most likely to be trivial, and as such from a practical perspective, the current evidence does not warrant general guidance on OCP use compared with non-use.
Exercise performance appeared relatively consistent across the OCP cycle, suggesting that different guidance is not warranted for OCP-taking days versus non-OCP taking days.
In the case of sportswomen who are focussing on performance, it is recommended that an individualised approach is sought, based on each athlete’s response to OCP use.

## Introduction

Sex hormones are one of the main determinants of biological sex [1]. During adulthood, levels of testosterone, the predominant male sex hormone, remain consistent in men [2], whilst concentrations of oestrogen and progesterone, the prevailing female sex hormones, undergo circamensal changes in women [3], marking one of the major differences between sexes. Moreover, the eumenorrheic menstrual cycle is susceptible to internal (e.g*.*, amenorrhea, oligomenorrhea and menorrhagia) and external (e.g., hormonal contraceptives) perturbations, highlighting the diversity in ovarian hormone profiles between women. In a recent audit of 430 elite female athletes, Martin et al. [4] showed that 213 athletes were hormonal contraceptive users, meaning that almost half of the population surveyed did not have a eumenorrheic menstrual cycle. Of these, 145 (68%) athletes reported taking oral contraceptive pills (OCPs), making them the most common type of hormonal contraceptive used and the second most common hormonal profile, after non-hormonal contraceptive users. These differences in endocrine profiles, between men and women, and amongst women (i.e., hormonal contraceptive users and non-users), highlight the need for sex-specific consideration within sport and exercise science.

Combined OCPs significantly reduce endogenous concentrations of 17 beta oestradiol and progesterone [5], when compared to the mid-luteal phase of the menstrual cycle, a stage when endogenous oestradiol and progesterone are relatively high. The exogenous oestrogens and progestins act via negative feedback on the gonadotrophic hormones, resulting in the chronic downregulation of the hypothalamic-pituitary-ovarian axis. Most combined, monophasic OCPs are second generation OCPs, containing low to standard doses of ethinyl oestradiol and either levonorgestrel, norethisterone, desogestrel or gestodene, delivered in a fixed amount every day for 21 OCP taking days (i.e.*,* consumption phase), followed by 7 OCP free days (i.e., withdrawal phase) [6]. In some countries, rather than a consumption and withdrawal approach, there are 21 active OCP days and 7 inactive OCP days. There are many types of OCPs with different compositions and potencies; for a comprehensive overview of hormonal contraceptives and OCPs please see Elliott-Sale and Hicks [6]. Overall, OCP use results in four distinct hormonal environments: (1) a downregulated endogenous oestradiol profile of ≈ 60 pmol·L^−1^ for 21 days that rises during the 7 OCP free days to ≈ 140 pmol·L^−1^; (2) a chronically downregulated endogenous progesterone profile of ≈ 5 nmol·L^−1^; (3) a daily surge of synthetic oestrogen and progestin that peaks within 1 h after ingestion [from ≈ 2 to ≈ 6 pg·mL^−1^], with baseline values accumulating slightly from ≈ 2 to ≈ 3 pg·mL^−1^ over the 21 OCP-taking days; (4) 7 exogenous hormone-free days [7]. These profiles, reflecting OCP consumption and withdrawal, are referred to as pseudo-phases, as they are “artificial” phases in comparison with the phases of the physiological menstrual cycle.

Aside from fertility control, OCPs are also used to alleviate the symptoms of dysmenorrhoea and menorrhagia; reduce the occurrence of premenstrual tension, symptomatic fibroids, functional ovarian cysts and benign breast disease; and decrease the risk of ovarian and endometrial cancer and pelvic inflammatory disease [8]. Furthermore, athletic populations have reported strategically using OCPs to manipulate the timing of, or omit entirely, the often-perceived inconvenient withdrawal bleed that occurs during the 7 OCP free days, using back-to-back OCP cycles [4, 9, 10]. Reliable and reversible contraception, along with the means to alleviate the side-effects associated with the eumenorrheic menstrual cycle, such as cramps/pain, bloating and headaches, and the ability to eliminate unpredictable menstruation, make OCPs a desirable option for many athletes.

Despite the prevalence of OCP use in athletic populations [4], the effects of OCPs on exercise performance are poorly understood. Although many experimental studies [11–13], numerous narrative and systematic reviews [14, 15] and books [16, 17] have addressed this topic, few in the area of sport and exercise science (e.g*.,* athletes, coaches, practitioners or researchers) truly understand the implications of OCP use on exercise performance, as previous research has shown conflicting findings on the directional effects of OCPs on outcomes such as muscle function [18, 19], aerobic and anaerobic [20–22] capacity and performance-based tests [23, 24]. As such, it is not possible to provide useful guidance to either the sporting or research community on how to work with athletes or participants using OCPs. Accordingly, the aim of this review was to investigate the effects of OCP use on exercise performance in women by making a between group comparison of OCP users and non-users (i.e.*,* naturally menstruating counterparts) and a within group comparison of OCP consumption and withdrawal. This is the first meta-analysis on the effects of OCPs on exercise performance. Additionally, this review is the first of its kind to appraise the quality of previous studies using robust assurance tools.

## Methods

### Design

The review was designed in accordance with the Preferred Reporting Items for Systematic Reviews and Meta-Analyses (PRISMA; Electronic Supplementary Material Appendix S1) guidelines [25], and consideration of the Population, Intervention, Comparator, Outcomes and Study design (PICOS, Table 1) was used to determine the parameters within which the review was conducted.

**Table 1:** Population, intervention, comparator, outcomes and study design (PICOS) criteria

Population	Healthy women aged 18–40 years were considered for inclusion in this study. No restrictions on activity level or training status were placed
Intervention	All participants were required to take an OCP, either habitually or experimentally. “Habitual” was defined as OCP use prior to the commencement of the study and not for the purposes of the study. “Experimentally” was defined as starting OCP use for the purposes of the study. All forms of OCPs were considered for use within this review
Comparator	Four broad types of comparisons were considered: (1) Between group comparison of habitual OCP users to naturally menstruating women. Women were phase matched in two ways for this comparison: (i) OCP withdrawal versus the early follicular phase of the menstrual cycle and (ii) OCP consumption versus all other phases of the menstrual cycle except for the early follicular phase; (2) within group comparison of OCP consumption with the hormone-free withdrawal phase; (3) comparison of active OCP use with non-use (e.g* .*, within-group comparison of women who were habitual users or non-users who stopped/started taking OCP for the purpose of the study); (4) randomised controlled trials of OCPs versus placebo intake ( e.g* .,* between group comparison of naturally menstruating women who were randomly assigned to either an OCP or placebo pill)
Outcomes	The primary outcome was to determine any differences in exercise performance, based on the comparisons described above. ‘Exercise performance’ referred to outcomes stemming from: workload, time to completion and exhaustion, mean, peak outputs, rate of production and decline and maximum oxygen uptake (a full list of considered outcomes can be found in Table 2). Although maximum oxygen uptake is not a performance test, this physiology-based outcome was included as it is widely used as an indicator of performance and is often used to describe the fitness of participants. Different exercise outcomes, broadly categorised as endurance and strength were considered. All exercise outcomes were extracted, and effect size duplication of multiple outcomes from the same test accounted for within the statistical analysis, as described in Sect. 2.4
Study design	Any study design that included the information described above was considered for inclusion

*OCP* oral contraceptive pill

**Table 2:** Overview of studies included in the systematic review and meta-analysis

Study	Aim	Participant health and training status	Study design	Oral contraceptive pill type	Eumenorrheic group description	Exercise outcomes	Quality rating
Anderson et al. [35]	To measure the influence of exogenous, endogenous and low oestrogen conditions, on contraction-induced muscle damage in young women	Healthy women (24.8 ± 2.3 years) who were not involved in a structured resistance program, or progressive and intense aerobic program during, or within the 6 months prior, to the study	Parallel group, observational, single measure	Monthly ethinyl oestradiol-containing OCP	Women with a self-reported natural monthly MC, tested at the EF and ML phases, verified using MC history, counting of days and serum oestrogen levels	Maximal voluntary isometric contraction of the leg extensor (N)—S	Low
Armstrong et al. [36]	To measure the influence of different methods of exogenous hormonal contraceptive (OCP, injectable steroid contraceptive, or no contraceptive) on thermal, metabolic, cardiorespiratory, performance, body composition and perceptual response of healthy young women (contraceptive) to a 7–8 week program of heat acclimation and physical training	Healthy women (21 ± 3 years) who were not undertaking frequent physical training	Parallel group, intervention, repeated measures	Oral ethinyl oestradiol and progestin contraceptives (Ortho-Novum, Ortho-Cyclen, Northi-TriCyclen, Marvelon or Femodene)	Women with a self-reported natural monthly MC, tested at the EF phase, verified by serum oestrogen and progesterone levels	$\dot{V}$O_2_ peak (ml·kg·min^−1^) measured during an incremental run to volitional fatigue—E	Low
Bell et al. [37]	To measure the influence of OCP on hamstring neuromechanics and leg stiffness across the MC	Healthy women (20.2 ± 1.4 years) who were physically active (defined as a minimum of 20 min of activity three times per week)	Parallel group, observational, repeated measures	Monophasic OCP	Women with a self-reported natural monthly MC for the previous 6 months, tested at the EF and ovulation phase, verified using urinary ovulation detection and serum oestrogen and progesterone levels	Rate of force production (N·s^−1^), and time to reach 50% peak (ms) measured during a maximal voluntary isometric hamstring contraction—S	Moderate
Bemben et al. [38]	To measure the influence of OCP on growth hormone and prolactin responses and on energy substrate utilization during prolonged submaximal exercise	Healthy, moderately active women (25.1 ± 1.4 years)	Parallel group, observational, single-measure	Multi or monophasic OCPs containing 35 µg of oestrogen (Ortho Novum 10/11, 7–7–7, 1/35 and Demulen)	Women with a self-reported natural monthly MC (cycles ranging from 28 to 35 days in length), for one year prior to the study, tested at the EL, ML and LL phases, verified by BBT and serum progesterone	$\dot{V}$O_2_ peak (ml·kg·min^−1^) and absolute workload (m·min^−1^) measured during an incremental run to volitional fatigue—E	Low
Bushman et al. [39]	To measure the effect of menstruation and OCP on power performance	Healthy, moderately active women (21.6 ± 2.6 years)	Parallel group, observational, repeated measures	2 participants took a monophasic and 15 a multiphasic OCP	Women with a self-reported natural monthly MC tested at the EF and EL phases, verified by BBT and urinary ovulation detection test	Estimated $\dot{V}$O_2_ peak (ml·kg·min^−1^) measured from the Forestry Step Test—E; peak power (W or W·kg^−1^), anaerobic capacity (W or W·kg^−1^) and power decline (W or W·kg^−1^) measured by the Wingate test—E and anaerobic power (kgm·s^−1^) measured in the Margaria Kalamen test—E	Low/very low
Casazza et al. [20]	To measure the effects of MC phase and triphasic OCP use on peak exercise capacity	Healthy, habitually active women who were not competitive athletes (25.5 ± 1.5 years)	Within group, intervention (OCP), repeated measures	Standardized triphasic OCP (days 1–7: 0.035 mg ethinylestradiol and 0.18 mg norgestimate; days 8–14: 0.035 ethinylestradiol and 0.215 norgestimate; days 15–21: 0.035 mg ethinylestradiol and 0.25 mg norgestimate, days 22–28: placebo pill)	Women with a self-reported natural monthly MC (22–32 days in length) for at least 6 months, tested during the LF and ML phases, verified by a urinary ovulation detection test and serum oestrogen and progesterone	Peak $\dot{V}$O_2_ (L·min^−1^), power (W) and time to exhaustion (min) measured during an incremental cycle to volitional fatigue—E	Moderate
de Bruyn-Prevost et al. [40]	To measure the effects of OCP and eumenorrheic MC on the physiological response to aerobic and anaerobic endurance tests	Women (22 ± 2.2 years)	Parallel group, observational, repeated measures	No information	Women with a self-reported natural monthly MC, tested during the EF, ovulatory and LL phases, verified by BBT	$\dot{V}$O_2_ peak (L·min^−1^) and working capacity at a heart rate of 170 bpm (W) measured using an incremental cycle to volitional fatigue—E, and maximal pedal time (s) during a fixed load (350 W) anaerobic endurance test—E	Very low
Drake et al. [41]	To measure the effect of OCP and eumenorrheic MC on electromyography and mechanomyography during isometric muscle contractions	Healthy women (24 ± 1 years) who were not involved in an exercise program	Parallel group, observational, repeated measures	No information	Women with a self-reported natural monthly MC (26–32 days in length) tested at the EF, LF, ovulation and EL, verified using urinary ovulation detection test	Maximal and submaximal isometric extensor and flexor contraction at 100, 75, 50 and 25% of maximal torque (N m)—S	Very low
Ekenros et al. [42]	To measure the effect of OCP and eumenorrheic MC on muscle strength and hop performance	Healthy women (26.7 ± 3.8 years) who were engaged in moderate to high levels of recreational activity	Within-group, intervention, repeated measures	Low dose monophasic OCPs containing ethinyl oestradiol (20–35 μg) combined with different progestogen (Levonorgestrel, Norgestimate, Drospirenone, Desogestrel, Noretisterone and Lynestrenol)	Women with a self-reported natural monthly MC who had not been taking any hormone-containing contraceptive for at least three months prior to the study, tested during the EF, ovulatory and ML phases, verified using urinary ovulation detection test and serum oestrogen and progesterone	Peak isokinetic knee extensor strength (N m)—S, handgrip strength (kg)—S and jump height during the one leg hop test (cm)—S	Moderate
Elliott et al. [5]	To measure the effect of OCP and MC on maximum force production	Healthy women (22 ± 4 years) who were sedentary (defined as not being involved in a strength or aerobic training program for the previous 6 months)	Parallel group, observational, repeated measures	Combined monophasic OCPs (Microgynon, Brevinor, Ovarnette, Marvalon, Cilest)	Women with a self-reported natural monthly MC (mean cycle length of 29 days) who were not taking any hormonal based contraction for 6 months prior to the study, tested during the EF and ML phases, verified by BBT, urinary ovulation detection test and serum oestrogen and progesterone	Maximal voluntary isometric force of the first dorsal interosseus muscle (N)—S, isokinetic extension and flexion of the quadriceps and hamstring muscles at 1.04. 2.09 and 4.19 rad/S (N m)—S, and isometric extension and flexion (N m)—S	Moderate
Giacomoni and Falgairette [43]	To measure the effect of time of day and OCP use on maximum anaerobic power	Physical education students (22.8 ± 2.8 years)	Parallel group, observational, repeated measures	Combined monophasic OCP (0.02–0.03 mg ethinylestradiol and 0.150 mg desogestrel or 0.075 mg gestodene)	Women with a self-reported natural monthly MC lasting 25–31 days in length, who had not used any OCP for at least 4 months before entering the study, tested during the LF and ML, verified by serum oestrogen and progesterone levels	Peak velocity (rpm)—E, peak force (kg)—S and peak power (W)—E, measured during a force velocity test	Moderate
Giacomoni et al. [22]	To measure the effect of OCP and eumenorrheic MC on anaerobic performance	Physical education students (23 ± 3 years)	Parallel group, observational, repeated measures	Combined monophasic OCP with constant oestrogen and progesterone levels (0.02–0.03 mg ethinylestradiol and 0.150 mg desogestrel or 0.075 mg gestodene)	Women with a self-reported natural monthly MC lasting 25–31 days in length, who had not used any OCP for at least 4 months before entering the study, tested during the LF and ML, verified by serum oestrogen and progesterone levels	Peak velocity (rpm)—E, peak force (kg)—S and peak power (W)—E, measured during a force velocity test and jump height (cm) measured using multi and squat jump tests—S	Moderate
Gordon et al. [44]	To measure the effect of OCP and MC on peak isokinetic torque	Healthy, well-trained women (20.6 ± 1.2 years)	Parallel group, observational, repeated measures	Monophasic OCP	Women with a self-reported natural monthly MC (mean cycle length of 28 days) tested during the EF, LF, ML and LL phases, verified by salivary oestrogen and progesterone levels	Peak concentric knee flexor and extensor torque at 60, 120, 18- and 240° (N m)—S	Very low
Gordon et al. [45]	To measure the effect of OCP and eumenorrheic MC on incidence of $\dot{V}$O_2_ max plateau and associated cardiorespiratory dynamics	Healthy, physically active women (21 ± 1.8 years)	Parallel group, observational, repeated measures	Monophasic OCP containing 30 µg ethinyl oestradiol and 150 µg levonorgestrel	Women with a self-reported natural monthly MC tested during the EF, LF, ML and LL, verified by MC history and salivary oestrogen and progesterone levels	Peak $\dot{V}$O_2_ (L·min^−1^) and power (W) measured during an incremental run to volitional fatigue—E	Moderate
Grucza et al. [46]	To measure the effect of OCP and eumenorrheic MC on thermosensitivity	Healthy women (21.3 ± 1.8 years) who were undertaking approximately 2–3 h of various activity types per week	Parallel group, observational, repeated measures	Monophasic OCP (Trikvilar or Neo-Gentrol 150/30)	Women with a self-reported natural monthly MC for one year preceding the experiment and who had never taken OCPs, tested during the LF and ML phase, verified by BBT	$\dot{V}$O_2_ peak (ml·kg·min^−1)^ measured during an incremental cycle to volitional fatigue—E	Low
Grucza et al. [47]	To measure the effect of OCP and eumenorrheic MC on cardiorespiratory responses to exercise	Healthy university students (21.3 ± 1.8 years)	Parallel group, observational, repeated measures	Monophasic OCP (Trikvilar or Neo-Gentrol)	Women with a self-reported natural monthly MC for 1 year preceding the experiment and who had never taken OCPs, tested during the LF and ML phase, verified by BBT	$\dot{V}$O_2_ peak (ml·kg·min^−1^) measured during an incremental cycle to volitional fatigue—E	Low
Hicks et al. [48]	To measure the effect of OCP and eumenorrheic MC on exercise induced muscle damage, and tendon properties	Healthy, recreationally active women (22.3 ± 2.3 years)	Parallel group, intervention, repeated measures	Combined monophasic OCP with ethinyl oestradiol dosage between 20 and 30 µg	Women with a self-reported natural monthly MC (average cycle length of 28 days) and who had never taken the OCP, tested during the ovulatory phase, verified by serum oestrogen	Peak voluntary isometric torque (N m)—S	Moderate
Isacco et al. [49]	To measure the effect of OCP and eumenorrheic MC on lipid oxidation and cardiorespiratory parameters at the anaerobic threshold and maximum capacity	Weight stable, healthy women (22 ± 2.9 years) who were recreationally active (defined as those not involved in any regular exercise training)	Parallel group, observational, repeated measures	Low-dose monophasic OCP contained 20 (* n* = 8) or 30 (* n* = 3) µg of ethinylestradiol and gestodene or levonorgestrel	Women with a self-reported natural monthly MC (average cycle length of 28 days for at least 1 year) and had not taken any OCP for more than 1 year prior to the study beginning, tested during the ML phase, verified by counting of days and serum oestrogen and progesterone levels	$\dot{V}$O_2_ peak (ml·kg·min^−1^) measured during an incremental cycle to volitional fatigue—E	Moderate
Joyce et al. [13]	To measure the effect of long-term OCP use on endurance performance	Healthy women (21 ± 2.7 years) who were recreationally active (defined as exercising > 3 days per week for at least 30 min per session)	Parallel group, observational, single measure	Combined monophasic OCP	Women with a self-reported natural monthly MC lasting between 28 and 30 days for at least 12 months before the study, tested during the EF phase, verified by serum oestrogen and progesterone levels	Peak $\dot{V}$O_2_ (L·min^−1^) and power (W) measured during an incremental cycle to volitional fatigue—E, and time to exhaustion (s) on a submaximal cycling test—E	Moderate
Joyce et al. [50]	To measure the effect of sex and OCP on submaximal cycling performance following an eccentric exercise protocol	Healthy women (20.8 ± 2.4 years) who were regularly physically active, but not participating in any regular resistance-exercise training	Parallel group, intervention, repeated measures	Combined monophasic OCP	Women with a self-reported natural monthly MC lasting between 28 and 30 days for at least 12 months before the study, tested during the EF phase and verified serum oestrogen and progesterone levels	Peak $\dot{V}$O_2_ (ml·kg·min^−1^) and power (W) measured during an incremental cycle to volitional fatigue—E, and mean torque (N m·kg^−1^) and torque decline (N m) measured across 240 maximal eccentric quadriceps contractions—S	Low
Lebrun et al. [23]	To measure the effect of OCP and eumenorrheic MC on exercise performance in highly active women	Healthy, athletic women (18–40 years), but none that competed in aerobic activities (cycling, triathlon, rowing, cross-country skiing)	Randomised controlled trial	Triphasic OCP (Synphasic, 0.035 mg ethinylestradiol and 0.5–1.0 mg norethindrone)	Women with a self-reported natural monthly MC (24–35 days in length) and no OCP use in the 3 months before entering the study, tested during the EF and ML phases, verified by serum oestrogen and progesterone levels	$\dot{V}$O_2_ peak (L·min^−1^) measured during an incremental cycle to volitional fatigue—E, time to exhaustion (s) in a submaximal endurance test—E, time to exhaustion (s) in an anaerobic speed test—E and peak quadriceps and hamstring torque (N m)—S	Moderate
Lee et al. [51]	To measure the effect of OCP and eumenorrheic MC on anterior cruciate ligament elasticity, force to flex the knee and knee flexion–extension hysteresis	Healthy, non-athletic women (24.7 ± 2 years)	Parallel group, observational, repeated measures	Low dose OCP containing < 50 µg ethinyl-estradiol	Women with a self-reported natural monthly MC for at least 6 months, with an average cycle length of 29 days, tested during the EF, LF, ovulatory and ML phases, verified by serum oestrogen and progesterone levels	Knee flexion force (N)—S	Moderate
Lynch and Nimmo [52]	To measure the effect of OCP and eumenorrheic MC on intermittent exercise performance	Healthy women (25.3 ± 6 years) who were recreationally active but not training for any one sport exclusively	Parallel group, observational, repeated measures	Low-dose monophasic OCP (Femodene, Cilest, Ovranette, Microgynon)	Women with a self-reported natural monthly ovulatory MCs with an average cycle length of 29 days, and who had either never taken OCPs or had not taken an OCP in the last 4 months, tested during the LF and LL phases, verified by serum progesterone levels	$\dot{V}$O_2_ peak (ml·kg·min^−1^) measured during an incremental run to volitional fatigue—E, and time to exhaustion (s) in an intermittent sprint test—E	Moderate/ low
Lynch et al. [53]	To measure the effect of OCP on performance and metabolic responses to, intermittent exercise during the 1^st^ or 3^rd^ week of the OCP cycle	Healthy, untrained women (23.1 ± 4 years)	Single group, observational, repeated measures	Low dose monophasic OCP (Ovranette, Femodene, Mercilon, Microgynon, Brevinor)	N/A	Time to exhaustion (s) in the final sprint of an intermittent sprint protocol—E	Moderate
Mackay et al. [67]	To measure the effect of OCP use on indirect markers of muscle damage following eccentric cycling in women	Healthy women (27.7 ± 4.5 years) who were not actively participating in any resistance or flexibility training in the 6 months prior to the study	Parallel group, acute intervention, single measure	Third and fourth generation monophasic OCP (ethinyl estradiol 0.02 µg; drospirenone 3 µg)	Women with a self-reported natural monthly MC (between 24 and 35 days) and who were not using any form of hormone-based contraceptive methods for 6 months prior to the study, tested during the ovulatory phase, verified by urinary ovulation detection kit and salivary oestrogen and progesterone levels	$\dot{V}$O_2_ peak (ml·kg·min^−1^) measured during an incremental cycling test to volitional fatigue—E, maximal voluntary knee extensor contraction at 90% knee flexion (N)—S, and mean power (W) during an eccentric cycling test—E	High/ moderate
Mattu et al. [68]	To measure maximal and submaximal exercise outcomes at different phases of the menstrual and OCP cycle	Healthy, trained, women (25.5 ± 5.2 years) who performed moderate to vigorous physical activity at least 4 times per week, and for at least 30 min per bout	Parallel group, observational, repeated measures	Second or third generation monophasic OCP containing between 20 and 35 µg of ethinyl oestradiol and 100–200 µg of progestin)	Women with a self-reported natural monthly MC (cycle between 21 and 35 days in length) who were non hormonal contraceptive users for at least 12 months prior to the study, tested during the LF and ML phases, tested using urinary ovulation detection test	$\dot{V}$O_2_ peak (L·min^−1^ or ml·kg·min^−1^) during an incremental ramp test to volitional fatigue—E, and time to exhaustion (s) during a constant load test at 85% peak power—E	High
Minahan et al. [54]	To measure the effect of sex and OCP in the response to muscle damage after intense eccentric exercise	Healthy women (21 ± 2.7 years) who were habitually active (primarily moderate intensity endurance-based activities), but who were not undertaking a resistance training program	Parallel group, intervention, repeated measures	Combined monophasic OCP	Women with a self-reported natural monthly MC that occurred every 28–30 days, tested during the EF phase, verified by serum oestrogen levels	Peak and mean isometric torque (N m and N m·kg^−1^) across 240 eccentric contractions—S	Low
Minahan et al. [55]	To measure the effect of OCP and the eumenorrheic MC on core body temperature and skin blood flow at rest and during exercise (temperate and hot environments)	Healthy women (22 ± 3.4 years) who were recreationally active (300–500 min per week of moderate intensity exercise)	Parallel group, observational, repeated measures	Low dose combined monophasic OCP	Women with a self-reported natural monthly MC (every 25–32 days) for more than 12 months and who had never taken any form of synthetic hormones, tested during the EF phase, verified by serum oestrogen and progesterone levels	Peak $\dot{V}$O_2_ (ml·kg·min^−1^) and power (W) measured during an incremental cycle to volitional fatigue—E, and mean power output (W) during a 3-stage submaximal test—E	Moderate
Ortega-Santos et al. [56]	To measure the effect of OCP and eumenorrheic MC on substrate oxidation during steady-state exercise	Healthy trained women (35.6 ± 4.2 years) who were training in either endurance or strength activities for 5–12 h per week	Parallel group, observational, repeated measures	Stable monophasic	Women with a self-reported natural monthly MC tested during the EF, LF and ML phase, verified by MC history and serum oestrogen and progesterone	$\dot{V}$O_2_ peak (ml·kg·min^−1^) measured during an incremental run to volitional fatigue—E	Low
Peters and Burrows [57]	To measure the effect of the androgenicity of progestins in OCP on leg strength	University athletes (20.2 ± 0.5 years) from a variety of sports (cricket, football, endurance running and swimming)	Parallel group, observational, repeated measures	Monophasic OCP containing 30 µg ethinylestradiol with 120 µg levonorgesterel or 250 µg norgestimate	N/A	Peak leg extension and flexion torque (N m)—S	Moderate
Quinn et al. [58]	To measure the effect of long-term OCP use on cerebral oxygenation during incremental cycling to exhaustion	Healthy women (21 ± 3 years) who were recreationally-active (defined as 150–300 min per week of moderate intensity exercise)	Parallel group, observational, single measure	28-day combined monophasic OCP	Women with a self-reported natural monthly MC (28–30 days in length) and had not taken any form of hormonal contraception for 12 months prior to the study, tested during the EF phase, verified by serum oestrogen and progesterone levels	Peak $\dot{V}$O_2_ (ml·kg·min^−1^) and power (W) during an incremental cycle to volitional fatigue—E	Moderate
Rebelo et al. [59]	To measure the effect of OCP on peak aerobic capacity and at the anaerobic threshold level in active and sedentary young women	Healthy women (23 ± 2.1 years), who were active (running or spinning 4–5 times per week) or sedentary (not engaging in regular physical activity for the previous 12 months)	Parallel group, observational, single measure	Monophasic OCP (0.2 mg ethinylestradiol and 0.15 mg gestodene)	N/A	Peak $\dot{V}$O_2_ (ml·kg·min^−1^) and power (W) during an incremental cycle to volitional fatigue—E	Moderate
Rechichi et al. [60]	To measure the effect of OCP cycle on endurance performance	Trained cyclists and triathletes (34 ± 7 years)	Single group, repeated measures, observational	Monophasic OCP (20–35 µg ethinylestradiol and 100–3000 µg progestin)	N/A	Mean power output (W) during a 1 h time-trial—E	High
Rechichi et al. [19]	To measure the effect of OCP cycle on common team sport performance variables	Team sport athletes (23.5 ± 4.5 years)	Single group, observational, repeated measures	Monophasic OCP (30 mcg ethinylestradiol with 150 mcg levonorgestrel, 2000 mcg cyproterone acetate, 3 mg drospirenone or 500 mcg norethisterone)	N/A	Jump height (cm) measured during a countermovement and a reactive strength (30 and 45 cm) jumps—S; 10 s cycle peak power (W·kg^−1^) and total work done (J·kg^−1^)—E; 5X6 second repeated sprint total work (J·kg^−1^) and power decrement (%)—E	High
Rechichi et al. [24]	To measure the effect of OCP cycle on 200 m swimming performance and associated measures of heart rate, blood lactate, pH and blood glucose	Competitive swimmers and water polo players (26 ± 4 years)	Single group, repeated measures, observational	Monophasic OCP (30 µg ethinylestradiol and 150 µg levonorgestrel)	N/A	Time to complete (s) a 200 m swim—E	High
Redman and Weatherby [61]	To measure the effect of OCP cycle on anaerobic performance	Elite and sub-elite rowers (20 ± 1.9 years)	Single group, repeated measures, observational	Combined triphasic OCPs (Triphasil-28)	N/A	Peak power output (W) during a 10 s maximal row—E, and time to complete (s) a 1000 m row—E	High
Sarwar et al. [18]	To measure the effect of eumenorrheic MC on muscle strength, contractile properties and fatigability in eumenorrheic and OCP users	Healthy, relatively sedentary women (20.6 ± 1.2 years)	Parallel group, observational, repeated measures	Combined (monophasic) OCPs with low dose ethinyl oestradiol (20–35 µg) together with progestins in different doses	Women with a self-reported natural monthly MC lasting between 26 and 32 days (mean cycle length of 28 days), tested during the EF, LF, ovulatory, ML and LL phase, verified by counting of days	Peak handgrip and quadricep strength (N)—S	Low
Schaumberg et al. [62]	To measure the effect of OCP use on peak physiological, cardiovascular and performance adaptations to sprint interval training	Healthy women (25.5 ± 5.4 years ) who were recreationally active, but not competitive at state or national level in any sport	Parallel group, intervention, repeated measures	Combined monophasic (20–30 µg ethinylestradiol and * n* = 5 androgenic, * n* = 5 anti-androgenic, and * n* = 15 non-androgenic progestins)	Women with a self-reported natural monthly MC, tested during the ML phase, verified by MC history, counting of days, urinary ovulation detection kit and serum oestrogen and progesterone levels	$\dot{V}$O_2_ peak (L·min^−1^) and peak power output (W) measured during an incremental cycle to volitional fatigue—E	High
Sunderland et al. [63]	To measure the effect of OCP and eumenorrheic MC on the growth hormone response to sprint exercise	Physically active women who regularly participated in repeated sprint type activities (21.5 ± 3.8 years)	Parallel group, observational, repeated measures	Monophasic OCP with high androgenicity (Microgynon, Ovranette, Mercilon, Loestrin)	Women with a self-reported natural monthly MC that varied in length from 27 to 35 days, tested during the LF and ML phase, verified by urinary ovulation detection test and serum oestrogen and progesterone levels	Mean and peak power output (W) during a 30 s treadmill sprint—E	Moderate
Vaiksaar et al. [64]	To measure the effect of OCP cycle on substrate use and lactate level over a 1 h submaximal rowing exercise	Trained rowers (21 ± 2.8 years)	Single group, observational, repeated measures	Monophasic OCP (20 μg ethinylestradiol and 75 μg gestodene)	N/A	$\dot{V}$O_2_ peak (L·min^−1^) measured from a maximal rowing test—E, and submaximal mean power output (W) measured during a submaximal rowing test—E	Moderate
Vaiksaar et al. [65]	To measure the effect of OCP and eumenorrheic MC on endurance performance	Recreational OCP users (21.0 ± 2.6 years), trained eumenorrheic (18.8 ± 2.1 years), recreational eumenorrheic (18.0 ± 0.9 years)	Parallel group, observational, repeated measures	Monophasic OCP (20 μg ethinylestradiol and 75 μg gestodene)	Women with a self-reported natural monthly MC (24–35 days), with at least 6 months of documented MC, tested during the LF and ML phases, verified by MC history and serum oestrogen and progesterone levels	$\dot{V}$O_2_ peak (ml·kg·min^−1^) and peak power (W) measured during a maximal rowing test—E	High
Wirth and Lohman [66]	To measure the effect of OCP and vitamin B6 supplementation on static muscle function	Women (18–33 years)	Parallel group, observational, repeated measures	No information provided	Women with a self-reported natural monthly MC (25–30 days in length) who had not used an OCP agent for a period of 1 year prior to the study. Tested during the LF and ML phases and verified by counting of days	Grip strength (kg) and endurance time (s) measured during a handgrip test—S	Very low

*OCP* oral contraceptive pill, *MC* menstrual cycle, *EF* early follicular, *LF* late follicular, *EL* early luteal, *ML* mid-luteal, *LL* late luteal, *BBT* basal body temperature, $\dot{V}$ *O*_*2*_ *peak* peak oxygen uptake, *E* endurance, *S* strength

### Study Search and Selection

PubMed, The Cochrane Central Register of Controlled Trials (CENTRAL), ProQuest and SPORTDiscus were systematically searched using the search terms “oral contraceptives” AND “athletic performance”; “sports performance”; “muscle”; “skeletal muscle”; “strength”; “force”; “muscular strength”; “muscular force”; “power”; “anaerobic”; “anaerobic power”; “anaerobic performance”; “anaerobic capacity”; “aerobic”; “aerobic capacity”; “aerobic power”; “aerobic performance”; “endurance”; “endurance capacity”; “endurance power”; “endurance performance”; “fatigue”; “recovery”. Searches were limited to humans, English, and females and no date restriction was applied. Only original research articles were considered for inclusion and review articles or conference abstracts were excluded. An example electronic search strategy for PubMed, including limits, can be found in Electronic Supplementary Material Appendix S2. All searches were conducted in January 2019 by KES. Three independent reviewers (KES, KLM and KMH) undertook a three-phase screening strategy: title and abstract, full-text screen and full-text appraisal. The search was updated in April 2020 using the same search criteria and screening strategy. These papers were subsequently included within the review and the meta-analysis was updated.

### Data Extraction and Quality Appraisal

Data were extracted by ED using a pre-piloted extraction sheet. When data were presented in graphical, and not in numerical format, DigitizeIt software (Version 2.3, DigitizeIt, Germany) was used to convert the data. The quality of each review outcome (defined as each of the statistical models undertaken) was assigned using a strategy based on the recommendations of the Grading of Recommendations Assessment Development and Evaluation (GRADE) working group [26]. This approach considers the quality of research outcomes in a systematic review according to five domains, namely risk of bias, directness, consistency, precision and evidence of publication bias. Risk of bias and directness were assessed at the individual study level with mode ratings used to categorise whole outcomes. The meta-analysis results were subsequently used to ascertain the consistency, precision and risk of publication bias for each outcome. Each individual study was initially appraised using a modified version of the Downs and Black Checklist [27], which was specifically tailored for use in this review (see Electronic Supplementary Material Appendix S3). The modified quality appraisal checklist comprised 15 outcomes, and had a maximum attainable score of 16, with all studies classified as being of high (H; 14–16), moderate (M; 10–13), low (L; 6–9) or very low (VL; 0–5) quality. The results of this assessment were used to assign an a priori quality rating to each outcome. This a priori rating was either maintained, or downgraded a level, based on the response to two questions that were considered key to the directness of the research design, i.e.*,* Question 1: was the natural menstrual cycle phase confirmed using appropriate biochemical outcomes? Question 2: was the type of OCP described to the level of detail required for categorisation or replication? With regards to Question 1, for studies with OCP groups only, biochemical confirmation was not deemed necessary, as OCP users do not have cyclical fluctuations in endogenous sex hormones, in which case the a priori score was maintained rather than downgraded. This rating was then either maintained, or downgraded another level based on whether the results obtained were consistent (determined by visual inspection of effect size estimates and the degree of credible intervals [CrI] overlap); precise (with outcomes downgraded if they were based on < 5 data points) and whether or not publication bias was evidence (determined using Egger’s test along with visual inspection of funnel plots as described in Sect. 2.4). The proportion of studies in each category was reported, with the mode considered to represent the overall quality rating for each individual review outcome. Two independent reviewers (KES and KMH) verified the data extraction and quality appraisal.

### Data Analysis

Data were extracted from studies comprising both between group and within group designs. Pairwise effect sizes were calculated by dividing mean differences by pooled standard deviations. At the study level, variance of effect sizes were calculated according to standard distributional assumptions [28]. All meta-analyses were conducted within a Bayesian framework enabling the results to be interpreted more intuitively compared to a standard frequentist approach through use of subjective probabilities [29]. With a Bayesian framework, dichotomous interpretations of the results of a meta-analysis with regards to the presence or absence of an effect (e.g*.*, with p values) can be avoided, and greater emphasis placed on describing the most likely values for the average effect and addressing practical questions such as the probability the average effect is beyond a certain threshold [29]. The Bayesian framework is also particularly suited to hierarchical models and sharing information within and across studies to improve estimates [29]. In the present meta-analysis, three-level hierarchical models were conducted to account for covariance in multiple outcomes presented in the same study [30]. Initial models were conducted including both strength and endurance outcomes with a regression coefficient assessing difference in the average effects. Where no evidence of a difference was identified, the model was re-run combining both categories of outcomes to increase data to better estimate model parameters. Given the expectation of relatively small effect sizes, an a priori threshold of ± 2 was identified for outliers. Primary analyses were completed with outliers removed but results also presented from the full complement of studies as sensitivity analyses. Additionally, sensitivity analyses were conducted on data obtained from studies categorised as “high” or “moderate” in quality. Inferences from all analyses were performed on posterior samples generated by Hamiltonian Markov Chain Monte Carlo with Bayesian 95% CrIs constructed to enable probabilistic interpretations of parameter values [29]. Interpretations were based on visual inspection of the posterior sample, the median value (ES_0.5_: 0.5-quantile) and 95% CrIs. Cohen’s [31] standard threshold value of 0.2 was used to describe effect size as small, and values between 0 and 0.2 were described as trivial. Analyses were performed using the R wrapper package brms, which was interfaced with Stan to perform sampling [32]. Convergence of parameter estimates was obtained for all models with Gelman–Rubin R-hat values below 1.1 [33]. Additional sensitivity analyses were conducted by restricting the analysis to studies that included exercise performance as the primary study outcome. Assessment of publication bias using Egger’s multilevel test with effect sizes regressed on inverse standard errors [34] identified no evidence of publication bias with median absolute intercept values less than 0.1 across all analyses.

### Rationale for Between Group Comparisons

For the between group analyses of habitual OCP users to naturally menstruating women, the OCP withdrawal phase [days 1–7] was compared with the early follicular phase [days 1–5] of the menstrual cycle and the OCP consumption phase [days 8–28] was compared with all phases of the menstrual cycle [days 6–28] except the early follicular phase [days 1–5]. The OCP withdrawal phase was compared with the early follicular phase as during the withdrawal phase OCP users experience a withdrawal bleed and during the early follicular phase of the menstrual cycle women experience menstruation. In addition, during both phases endogenous concentrations of oestrogen and progesterone are comparably low. During the remainder of the menstrual cycle, endogenous concentrations of oestrogen and progesterone change over time (e.g*.*, the mid-cycle peak in oestrogen and the mid-luteal rise in progesterone and oestrogen) and there is large variation in endogenous concentrations of oestrogen and progesterone as a result of different OCP formulations. As such, it is difficult to make meaningful comparisons during these phases and this could be considered a limiting factor of any meta-analysis making between group comparisons of naturally menstruating women and OCP users. To reduce the impact of this limitation, a sensitivity analysis was completed on the between group design data to better match the physiological menstrual cycle and OCP pseudo-phases. This was achieved by mapping days 1–5, 12–16 and 19–23 from both cycles, which correspond with the early follicular, ovulatory and mid-luteal phases in a natural menstrual cycle and represents the following hormonal profiles: low oestrogen and progesterone, high oestrogen and low progesterone and high progesterone and medium oestrogen. As such, this meta-analysis (1) compared the two most stable phases of the OCP and menstrual cycles in the first between group analyses; (2) compared the two least stable phases of the OCP and menstrual cycles in the second between group analysis; and (3) performed an additional sensitivity analysis to better match the OCP and menstrual phases.

## Results

### Study Characteristics

**Fig. 1:**
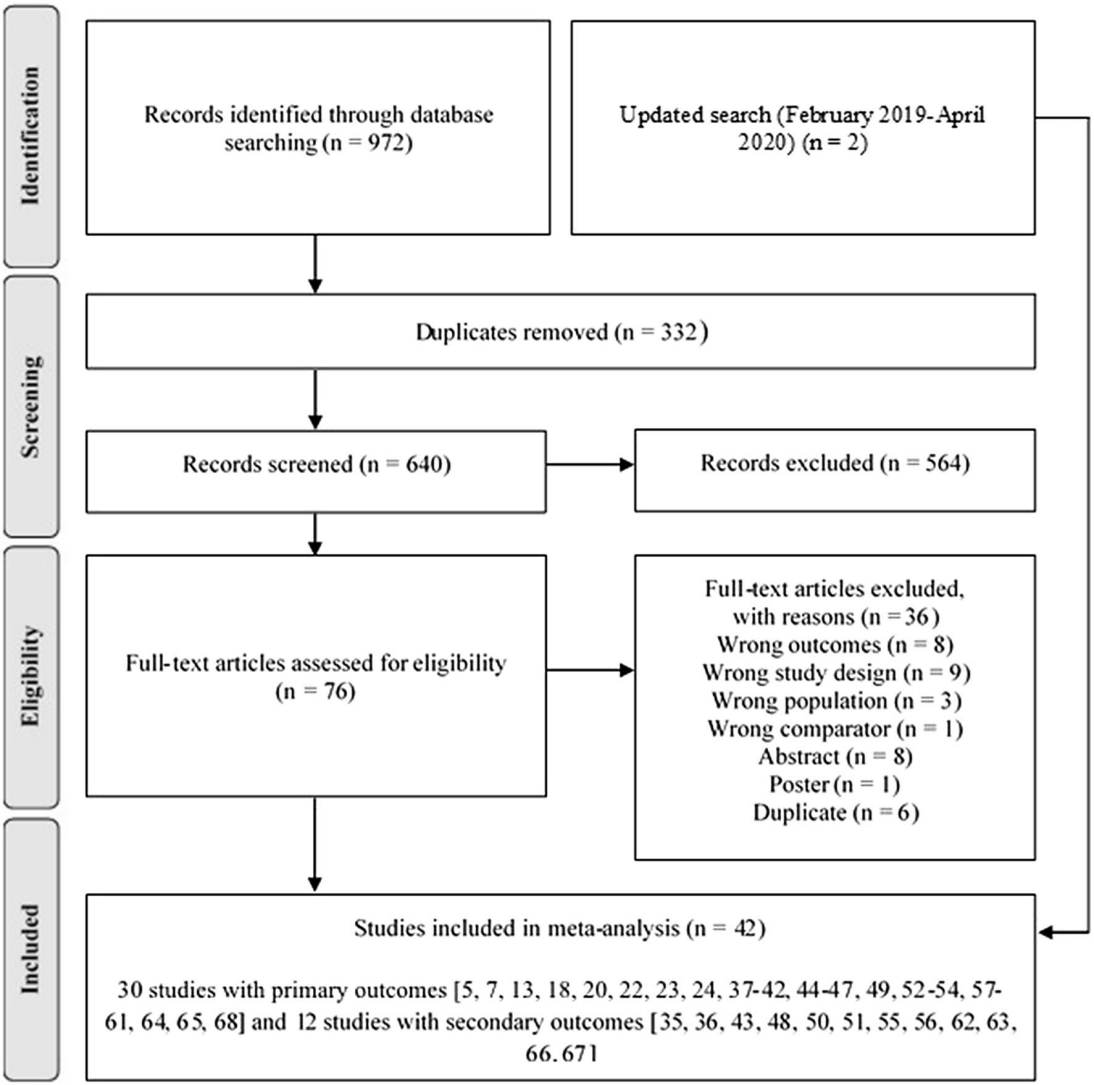
Search flow diagram

Figure 1 shows the studies identified and selected by the search strategy. Details of the included studies are shown in Table 2. In total 42 studies [5, 13, 18–20, 22–24, 35–68] and 590 participants were included.

**Fig. 2:**
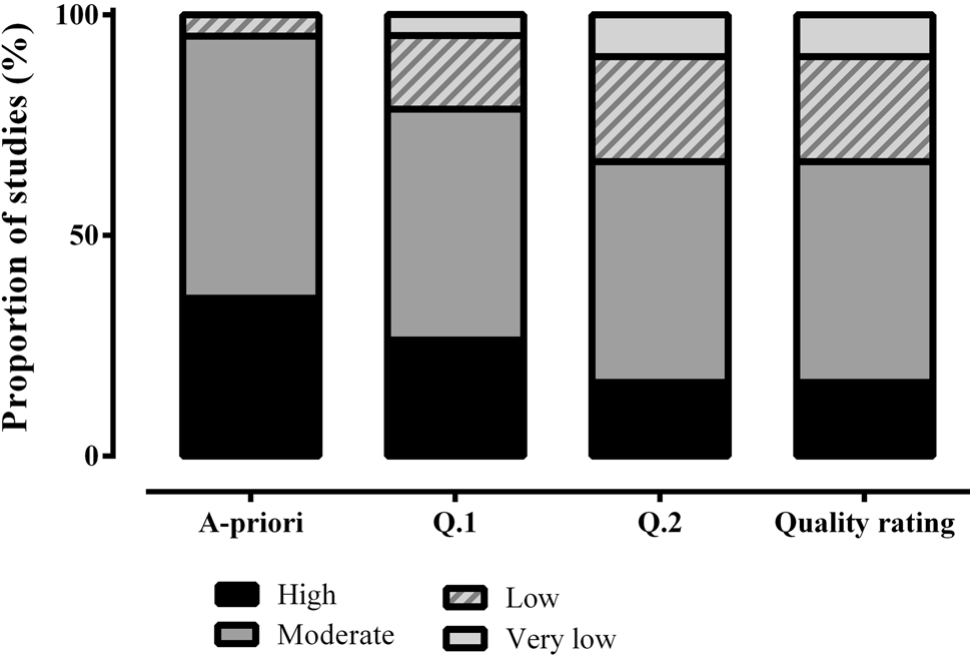
Quality rating of outcomes from all included studies (*n* = 42). Each bar represents the proportion of articles assigned a high, moderate, low, or very low-quality rating. The x-axis represents the different stages of this process, with the first bar based on the assessment of risk of bias and study quality as determined by the Downs and Black checklist, while question 1 (Q.1) and question 2 (Q.2) were used to determine if the natural menstrual cycle phase comparison was verified using appropriate biochemical outcomes and whether the oral contraceptive pill under investigation was described in a sufficient level of detail. The final bar represents the proportion of studies assigned to each quality rating category

Methodological quality at the level of the individual study is shown in Fig. 2; 83% of the studies were graded as M, L or very low VL, with 17% achieving H quality. Specifically, 4 studies were graded as VL, 10 as L, 21 as M and 7 as H quality.

### Between Group Analyses of Habitual Oral Contraceptive Users Compared to Naturally Menstruating Women

Thirty of the included studies (combined quality rating = M; specifically 20% H; 37% M; 30% L; 13% VL) generated 151 effects sizes from research designs comparing habitual OCP users with naturally menstruating women. The data were collected from 597 participants (habitual OCP *n* = 303, naturally menstruating *n* = 294) with studies comprising a mean group size of 10 (range *n* = 5–25).

#### Oral Contraceptive Pill Withdrawal [Days 1–7] Versus the Early Follicular Phase [Days 1–5] of the Menstrual Cycle

**Fig. 3:**
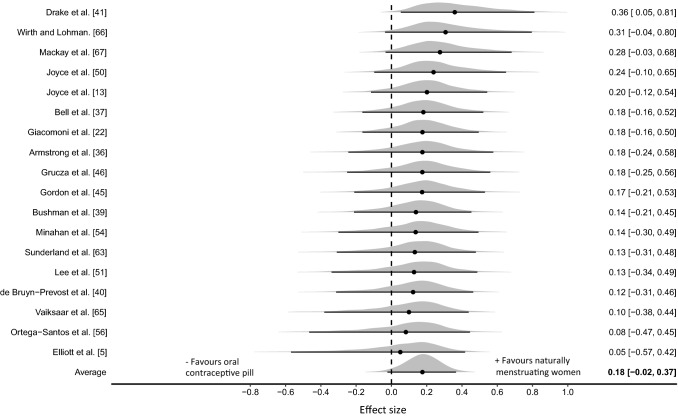
Bayesian Forest plot of multilevel meta-analysis comparing performance measured during oral contraceptive pill withdrawal phase and early follicular phase of the menstrual cycle. The study-specific intervals represent individual effect size estimates and sampling error. The circle represents the pooled estimate generated with Bayesian inference along with the 95% credible interval (95% CrI)

Three outliers were identified with effect sizes greater than + 2, and were removed from the analysis, leaving a total of 49 effect sizes (26 endurance, 23 strength) from 18 studies (combined quality rating = M; specifically 17% H; 33% M; 28% L; 22% VL; habitual OCP *n* = 176, naturally menstruating *n* = 169). The three-level hierarchical model indicated a trivial effect with the median value associating greater performances with naturally menstruating women (ES_0.5_ = 0.18 [95% CrI − 0.02 to 0.37]; Fig. 3). Relatively large between-study standard deviation was identified ($\tau$_0.5_ = 0.16 [95% CrI 0.01–0.44]) with estimates indicating moderate intraclass correlation (ICC_0.5_ = 0.42 [95% CrI 0.00–0.80]) due to analysis of multiple outcomes reported within studies. Pooling of strength and endurance outcomes was conducted as no evidence was obtained that indicated a differential effect between the performance categories (ES_0.5/Endurance-Strength_ = 0.04 [95% CrI  − 0.41 to 0.43]). Posterior estimates of the pooled effect size identified a moderate probability of a small effect favouring naturally menstruating women in the early follicular phase of the menstrual cycle (*d* ≥ 0.2; *p* = 0.404) and effectually a zero probability favouring habitual OCP women (*d* ≤  − 0.2; *p* = 0.001). Inclusion of outliers within the model substantially increased the average effect size (ES_0.5_ = 0.34 [95% CrI  − 0.04 to 0.72]) and between study variance ($\tau$_0.5_ = 0.70 [95% CrI 0.24–1.23]).

#### Oral Contraceptive Pill Consumption [Days 8–28] Versus all Phases of the Menstrual Cycle [Days 6–28] Except the Early Follicular Phase [Days 1–5]

**Fig. 4:**
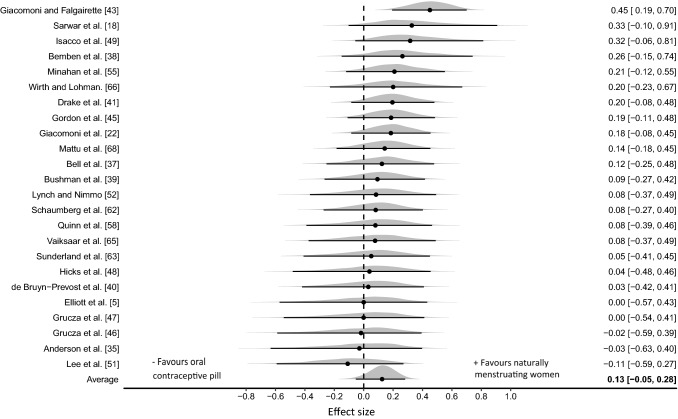
Bayesian Forest plot of multilevel meta-analysis comparing performance measured during oral contraceptive pill consumption phase with menstrual cycle phases (excluding early follicular phase). The study-specific intervals represent individual effect size estimates and sampling error. The circle represents the pooled estimate generated with Bayesian inference along with the 95% credible interval (95% CrI)

Eleven outliers were identified with effect sizes greater than + 2, and were removed from the analysis, leaving a total of 88 effect sizes (53 endurance, 35 strength) from 24 studies (combined quality rating = M; specifically 21% H; 42% M; 25% L; 13% VL; habitual OCP *n* = 244 habitual OCP, naturally menstruating *n* = 230). The three-level hierarchical model indicated a trivial effect with the median value associating greater performances obtained in the naturally menstruating women (ES_0.5_ = 0.13 [95% CrI  − 0.05 to 0.28]; Fig. 4). Relatively large between study variance was identified $\tau$_0.5_ = 0.22 [95% CrI 0.06–0.45] with central estimates indicating very low intraclass correlation ICC_0.5_ = 0.08 [95% CrI 0.0–0.61] due to analysis of multiple outcomes reported within studies. Pooling of strength and endurance outcomes was conducted as no evidence was obtained that indicated a differential effect between the performance categories (ES_0.5/Endurance-Strength_ = 0.02 [95% CrI  − 0.25 to 0.31]). Posterior estimates of the pooled effect size identified a small probability of a small effect favouring naturally menstruating women (*d* ≥ 0.2; *p* = 0.188) and effectually a zero probability favouring habitual OCP women (*d* ≤  − 0.2; *p* < 0.001). Inclusion of outliers within the model increased the average effect size (ES_0.5_ = 0.19 [95% CrI  − 0.14 to 0.51]) and between study variance ($\tau$_0.5_ = 0.71 [95% CrI 0.49–1.07]).

#### Sensitivity Analyses; Primary Outcome Studies/Moderate or High-Quality Studies only

Sensitivity analyses were completed for between and within group designs using data from studies that included exercise performance as the primary study outcome (Table 3) and from studies categorised as high or moderate in quality (Table 4). No substantive differences were obtained from any of the previous analyses with pooled effect sizes identifying trivial effects with greater performances obtained in naturally menstruating women.

**Table 3:** Results from sensitivity analyses with data from studies including performance as the primary outcome

Sensitivity analysis	Analysis details	Effect size	Between study variance	Intraclass correlation	Probability of small effect
Between group: oral contraceptive pill withdrawal versus the early follicular phase of the menstrual cycle	34 effect sizes from 11 studies (combined quality = H/M/L; 27% H; 27% M; 27% L; 18% VL)	0.14 [− 0.14–0.38]	0.20 [0.01–0.59]	0.28 [0.0–0.82]	(*d* ≥ 0.2; * p* = 0.323; * d* ≤ − 0.2; * p* = 0.014)
Between group: oral contraceptive pill consumption versus all phases of the menstrual cycle except the early follicular phase	57 effect sizes from 16 studies (combined quality = M; 26.7% H; 33.3% M; 26.7% L; 13.3% VL)	0.14 [− 0.03–0.31]	0.10 [0.0–0.40]	0.42 [0.0–0.86]	(*d* ≥ 0.2; * p* = 0.257; * d* ≤ − 0.2; * p* = 0.001)
Within group: oral contraceptive pill consumption with oral contraceptive pill withdrawal	141 effect sizes from 21 studies (combined quality = H/M; 33.3% H; 33.3% M; 19.1% L; 14.3% VL)	0.05 [− 0.03–0.11]	0.06 [0.0–0.17]	0.19 [0.0–0.66]	(|*d*|≥ 0.2; * p* < 0.001)

Results are from multilevel random effects models with median parameter estimates and 95% credible intervals (95% CrI)

*H* high, *M* moderate, *L* low, *VL* very low

**Table 4:** Results from sensitivity analyses with data from studies categorised as “high” or “moderate” in quality

Sensitivity analysis	Analysis details	Effect size	Between study variance	Intraclass correlation	Probability of small effect
Between group: oral contraceptive pill withdrawal versus the early follicular phase of the menstrual cycle	22 effect sizes from 9 studies	0.12 [− 0.24–0.43]	0.18 [0.01–0.61]	0.63 [0.0–0.88]	(*d* ≥ 0.2; * p* = 0.281; * d* ≤ − 0.2; * p* = 0.041)
Between group: oral contraceptive pill consumption versus all phases of the menstrual cycle except the early follicular phase	60 effect sizes from 15 studies	0.14 [− 0.09 to 0.33]	0.22 [0.05–0.48]	0.10 [0.0–0.55]	(*d* ≥ 0.2; * p* = 0.282; * d* ≤ − 0.2; * p* = 0.006)
Within group: oral contraceptive pill consumption with oral contraceptive pill withdrawal	89 effect sizes from 16 studies	0.03 [− 0.06 to 0.10]	0.04 [0.0–0.16]	0.38 [0.0–0.69]	(|*d*|≥ 0.2; * p* < 0.001)

Results are from multilevel random effects models with median parameter estimates and 95% credible intervals (95% CrI)

*H* high, *M* moderate, *L* low, *VL* very low

#### Sensitivity Analysis of Physiological Menstrual Cycle Phases Versus Pseudo Oral Contraceptive Pill Phases; Days 1–5, Days 12–16 and Days 19–23

An additional set of sensitivity analyses were completed on the between group design data to better match the physiological menstrual cycle and OCP pseudo-phases. This was achieved by mapping days 1–5, 12–16 and 19–23 from both cycles (Table 5). Collectively, findings were aligned with the more coarsely matched phases presented above (i.e., Sects. 3.2.1 and 3.2.2). In days 1–5 and 19–23, pooled effect sizes again identified trivial effects with greater performances obtained in naturally menstruating women. In days 12–16, pooled effect sizes were effectually zero with a wide CrI reflecting the limited data available (11 effect sizes from 5 studies).

**Table 5:** Results from sensitivity analyses comparing performance outcomes comparing physiological menstrual cycle phases versus pseudo oral contraceptive pill phases

Sensitivity analysis	Analysis details	Effect size	Between study variance	Intraclass correlation	Probability of small effect
Between group: days 1–5	42 effect sizes from 16 studies (combined quality rating = M; 18.75% H; 31.25% M; 25% L; 25% VL)	0.17 [− 0.04 to 0.38]	0.15 [0.01–0.50]	0.60 [0.10–0.90]	(*d* ≥ 0.2; * p* = 0.368; * d* ≤ − 0.2; * p* = 0.001)
Between group: days 12–16	11 effect sizes from 5 studies (combined quality rating = M; 60% M; 40% VL)	− 0.04 [− 0.73 to 0.58]	0.27 [0.01–1.28]	0.20 [0.10–0.70]	(*d* ≥ 0.2; * p* = 0.137; * d* ≤ − 0.2; * p* = 0.291)
Between group: days 19–23	38 effect sizes from 14 studies (combined quality rating = M; 28.6% H; 35.7% M; 21.4% L; 14.3% VL)	0.13 [− 0.13 to 0.34]	0.22 [0.02–0.56]	0.35 [0.01–0.65]	(*d* ≥ 0.2; * p* = 0.253; * d* ≤ − 0.2; * p* = 0.009)

Results are from multilevel random effects models with median parameter estimates and 95% credible intervals (95% CrI)

*H* high, *M* moderate, *L* low, *VL* very low

### Within Group Analyses of Oral Contraceptive Consumption with the Hormone-Free Withdrawal phase

**Fig. 5:**
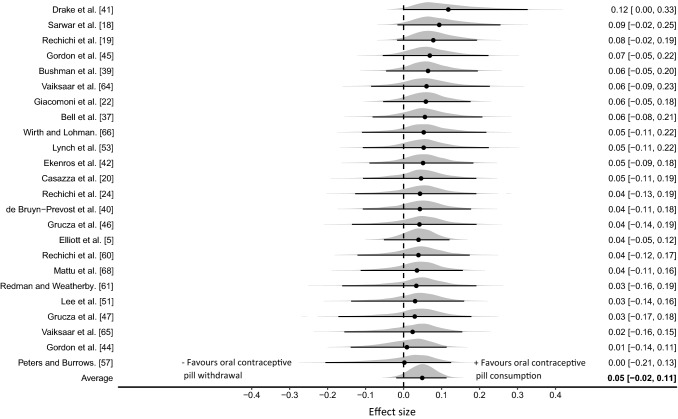
Bayesian Forest plot of multilevel meta-analysis comparing performance measured during oral contraceptive pill consumption with the hormone-free withdrawal phase. The study-specific intervals represent individual effect size estimates and sampling error. The circle represents the pooled estimate generated with Bayesian inference along with the 95% credible interval (95% CrI)

Twenty-four of the included studies (combined quality rating = H/M; specifically 33% H; 33% M; 17% L; 17% VL) generated 148 effect sizes (positive values favouring OCP consumption) from research designs comparing OCP consumption with OCP withdrawal. The data were collected from 221 participants with studies comprising a mean group size of 10 (*n* = 5–17). The three-level hierarchical model incorporating both strength (96 effect sizes) and endurance (52 effect sizes) provided some evidence of a trivial effect with the pooled effect size very close to zero (ES_0.5_ = 0.05 [95% CrI  − 0.02 to 0.11]; Fig. 5). Between study variance was relatively small $\tau$_0.5_ = 0.06 [95% CrI 0.0–0.16] as were central estimates of intraclass correlation ICC_0.5_ = 0.20 [95% CrI 0.0–0.62] due to analysis of multiple outcomes reported within studies. Pooling of strength and endurance outcomes was conducted as no evidence was obtained that indicated a differential effect between the performance categories (ES_0.5/Endurance-Strength_ = 0.02 [95% CrI  − 0.22 to 0.33]). Posterior estimates of the pooled effect size identified almost zero probability of a small effect in either direction (|*d*|≥ 0.2 *p* ≤ 0.001). Sensitivity analyses conducted with data from studies where performance was identified as a primary outcome had minimal effect on model outputs (Table 3) and from studies categorised as high or moderate in quality (Table 4) had no substantive influence on model outputs.

### Within Group Comparison of Oral Contraceptive Use and Non-Use

Only two studies [20, 42] met the inclusion criteria for this category and as such no meta-analysis was performed on these data. Casazza et al. [20] tested participants during two phases (4–8 days and 17–25 after the start of menses) of the menstrual cycle, in a randomised order. Following this, participants began taking the same triphasic OCP for four complete cycles (28 days per cycle) and were tested during the week of the inactive OCPs and during the second week of active OCP ingestion. Menstrual cycle phase had no effect on peak exercise capacity. Conversely, 4 months of OCP use resulted in significant decreases in time to peak exercise (14%) and the peak power output attained (8%) during a continuously graded cycle test. In addition, all participants experienced an 11% decline in peak oxygen uptake ($\dot{V}$O_2 peak_; L∙min^−1^). Ekenros et al. [42] employed a cross-over design, such that participants taking an OCP upon recruitment were tested on day 2, 3 or 4 during the OCP free days and on days 7 or 8 and 14 or 15 during the OCP-taking days, after which they stopped taking the OCP and were tested on day 2, 3 or 4, 48 h after ovulation and 7 or 8 days after ovulation. Those who were naturally menstruating at recruitment were tested on day 2, 3 or 4, 48 h after ovulation and 7 or 8 days after ovulation and were re-tested following one OCP cycle on day 2, 3 or 4 during the OCP free days and on days 7 or 8 and 14 or 15 during the OCP-taking days. There were no significant differences in muscle strength between groups, although maximum muscle strength of the knee extensors was different between the early follicular (days 2, 3 or 4) and luteal phase (7 or 8 days after ovulation) in the naturally menstruating group; 139 (28) N·m compared with 145 (26) N·m (*p* = 0.02).

### Randomised Controlled Trials of Oral Contraceptive Use Versus Placebo Intake

Only one study [23] met the inclusion criteria for this category and as such no meta-analysis was performed on these data. Lebrun et al. [23] employed a randomised, double-blind, placebo-controlled trial in naturally menstruating women. Testing was performed during the early follicular (days 3–8) and mid-luteal (days 4–9 after ovulation) phases of an ovulatory menstrual cycle, after which participants were randomly assigned to either an OCP (*n* = 7) or placebo (*n* = 7) group and were tested between days 14 and 17 of the second cycle of OCP (i.e., the same triphasic OCP) or placebo administration. Participants were active women, who regularly competed in aerobic activities such as running, cycling, triathlon, rowing, cross country skiing. OCP use resulted in a mean decrease of 4.7% in $\dot{V}$O_2max_ compared with a 1.5% improvement in the placebo group. The decrease in absolute $\dot{V}$O_2max_ was accompanied by an increase in the sum of skinfolds, but not by significant changes in weight or measures of strength, anaerobic, or endurance performance.

## Discussion

The aim of this review was to identify if OCP use influenced exercise performance. Results generally indicated a trivial performance effect on average with OCP use, with superior performance generally observed for naturally menstruating women compared to their OCP using counterparts. In addition to the estimated trivial to small average effect, results from the meta-analysis models indicated relatively large between study variance indicating that research design, participant characteristics and performance measured might influence any effect. Collectively, these findings indicate that OCPs might, on average, exert a slightly negative impact on performance, but from a practical point of view the effect magnitude and variability support consideration of an individual’s response to OCP use, so that decisions as to the appropriateness of OCP use can be tailored to the individual requirements (e.g*.*, contraceptive or medical need) and response (i.e., to what degree they might be affected) of each athlete. Pooling of data comparing exercise performance between OCP consumption and withdrawal estimated an effect that was very close to zero, indicating that exogenous supplementation of oestrogen and progestin is unlikely to have any substantive effect on exercise performance across an OCP cycle.

As a result of OCP use, endogenous concentrations of oestradiol and progesterone are significantly downregulated when compared with the mid-luteal phase of the menstrual cycle [5]. This chronic downregulation might be responsible for the slightly impaired exercise performance demonstrated in OCP users when compared with their naturally menstruating counterparts. Indeed, the endogenous hormonal profile of an OCP user is comparable to the profile observed during the early follicular phase of the physiological menstrual cycle; i.e., correspondingly low levels of endogenous oestradiol and progesterone [5, 69, 70]. In our meta-analysis [71], on the effects of the menstrual cycle on exercise performance, the available evidence indicated potentially inferior performance during the early follicular phase, when compared with all other phases of the menstrual cycle that had considerably higher concentrations of endogenous oestrogen and/or progesterone. Similarly, the within group results of the current meta-analysis showed that exercise performance between the OCP consumption and withdrawal phases was, on average, very unlikely to exhibit even a small effect, during which time the concentrations of endogenous oestradiol and progesterone were consistently low and did not significantly increase [5]. Collectively, these results indicate that exercise performance might be mediated by the concentration of endogenous ovarian hormones in some individuals, as reflected by evidence of slightly impaired performance on average at a time when these hormones are lowest.

The between-group findings from the present review align with those of Casazza et al. [20] and Lebrun et al. [23] who also showed that experimental OCP use resulted in reduced peak exercise capacity and decreased maximal oxygen uptake, when compared with non-hormonal contraceptive use. Casazza et al. [20] employed a cross-over design for their study, with data from two phases of a physiological menstrual cycle compared with data after 4 months of triphasic OCP use, whilst Lebrun et al. [23] utilised a randomised, double-blind, placebo-controlled trial, with data from two phases of the physiological menstrual cycle compared with data after 2 months of triphasic OCP use. These longitudinal intervention studies represent a change from inactive to active OCP use in the same individuals, which is a stronger research design when compared to the cross-sectional observational studies that were used in the between-group analysis in the present review, which further supports the notion that OCP use might result in small adverse effects on performance in some individuals when compared with naturally menstruating women. It is worth noting that experimental OCP use may not always be carried out in consultation with a clinician who would monitor any potentially unfavourable side effects, and possibly make changes to the OCP type or dose, as such higher detrimental effects may potentially be observed in experimental OCP users as opposed to habitual OCP users. In addition, some adverse side-effects, which are experienced during initial OCP use, can mitigate over time, potentially compounding the issue of comparing habitual OCP users with experimental OCP users.

Ekenros et al. [42] showed no difference in performance between OCP and non-OCP use, which is contrary to the findings from the present study and those of Casazza et al. [20] and Lebrun et al. [23]. Although Ekenros et al. [42] employed a longitudinal intervention study design, the original ‘non-OCP’ users only received a monophasic OCP for 1 month (i.e., 21 OCP-taking days) before they were retested as ‘habitual’ OCP users. Casazza et al. [20] and Lebrun et al. [23] retested after 4 and 2 months of OCP use, which might have resulted in a greater downregulation of endogenous oestradiol and progesterone than that seen by Ekenros et al. [42]. In addition, the participants in the Ekenros et al. [42] study used a variety of OCPs, whereas Casazza et al. [20] and Lebrun et al. [23] used the same OCP, resulting in a more homogenous group, with potentially less inter-individual variation in endogenous ovarian hormone concentration, and reducing the possibility of type II errors [72]. Ekenros et al. [42] used a strength based performance measure, whilst Casazza et al. [20] and Lebrun et al. [23] employed more endurance type performance measures, representing different physiological pathways for oestrogen and/or progesterone to exert their effects. For example, progesterone is likely to mediate changes in ventilatory drive [73], whilst oestrogen might be responsible for sex-differences in substrate metabolism [74], both considered to influence endurance performance. Whereas for strength-based performance, both sex hormones act as neurosteroids, which are capable of traversing the blood–brain barrier thereby potentially enacting effects on maximal neuromuscular performance [75]. These methodological differences, alongside the differing modes of exercise, might account for the disparity in result between Ekenros et al. [42] and Casazza et al. [20], Lebrun et al. [23] and the present review.

Our within group analysis indicates that the exogenous supplementation of ethinyl oestradiol and progestin is very unlikely to exert any substantive effect, such that performance was relatively consistent across an OCP cycle. From a practical perspective, this means that exercise performance is not moderated by the exogenous hormonal profile of an OCP but is more likely mediated by the endogenous hormonal milieu caused by OCP use (i.e.*,* the continuous downregulation of oestradiol and progesterone between OCP consumption and withdrawal). These data suggest that the ‘supplementary’ nature of OCPs should not be considered as performance-enhancing. As OCPs are also not ergolytic, the timing of the withdrawal bleed can be manipulated (e.g*.*, to avoid bleeding during competition) without negatively impacting performance, although the long-term health implications of continuous OCP consumption without any withdrawal are unknown. Schaumberg et al. [10] have noted that menstrual manipulation for exercise and sports performance reasons is already a fairly common practice amongst physically active women.

Although all results from the current meta-analysis align, and have solid mechanistic underpinnings, it is important to acknowledge that the practical implications of these findings are small. All point estimates and outliers were in the same direction and indicated a potentially negative influence, on average, of ovarian hormonal suppression on performance. However, the real-life implications of these findings are likely to be so small as to be trivial and therefore not meaningful for most of the population. Additionally, a large range of moderating factors [76, 77] (independent of hormonal changes) are likely to influence an individual’s response to, and requirement for, OCPs and we suggest that individuals do not solely make their decision to use or not use OCPs based on the performance related findings reported herein. For example, some individuals are prone to substantial menstrual symptoms such as cramps, bloating or heavy menstrual bleeding, and for these individuals, the benefits of OCP use [78, 79] might outweigh the small detriments observed in the present review. Similarly, the consequences of unplanned pregnancy might be far greater than the trivial effects observed in the current meta-analysis. Conversely, large inter-individual variation exists in the response to most interventions [80, 81] whereby some individuals might experience no performance-related side-effects whatsoever, whereas others might experience substantial performance-related side-effects from OCP use [4]. As such, we recommend that individuals consider all relevant factors (which might include physical, emotional, practical, financial and health related aspects) before making decisions as to the appropriateness (or not) of OCP use.

The current review was primarily conducted on non-randomised observational trials, which might be considered a limitation of its value. Randomised controlled trials are the preferred design to investigate the potential influence of a treatment (in this case OCPs) on an outcome (in this case exercise performance); however, they can be difficult to implement in this population, as individuals tend to be habitual OCP users or non-users. Only one randomised controlled trial was identified from the relevant literature [23], alongside two further trials wherein an OCP was prescribed to or withheld from non-users and habitual users in a cross-over design [20, 39]. Withholding OCPs from a habitual OCP user might have ethical and practical (e.g*.*, unplanned pregnancy) implications and as such, this type of research design is rarely employed. In addition, having the resources to conduct appropriately standardised and controlled studies across the time-periods required to adequately address this question is, in many cases, prohibitive (i.e., an adequate wash-out and/or supplementation period). Instead, most data on OCP use versus non-use are based on between group investigations of independent parties, which might be impacted by a large range of confounding variables and does not permit causal inference to be made. The lack of randomised controlled trials will affect analyses within this area of study for the foreseeable future.

Following the Downs and Black quality assessment [27], most studies (64%) were classified as M or L, which was largely due to a lack of standardisation (e.g*.*, prior activity and food intake) and inadequate familiarisation (i.e., often no familiarisation took place or long periods of time had elapsed between testing sessions, potentially warranting re-familiarisation). Additionally, most studies had small samples (range: *n* = 5–25), with a mean group size of 10, meaning that many were likely to be under-powered. Rigorous control of these research design factors in future studies, along with consideration of individual response [65, 66] and more randomised controlled trials will provide further insight into the effects of OCP use on exercise performance and will allow exercising women to make evidence-based decisions on OCP use within the context of sport. Moreover, consideration of the topic-specific methodological issues recommended by Cable and Elliott [82] and Elliott-Sale et al. [72], namely biochemical confirmation of menstrual phase and adequate description of OCP type, resulted in a further reduction in high quality studies, from 36 to 17%, and an increase in very low-quality studies, from 0 to 10%. Future studies should use appropriate biochemical outcomes (i.e., blood samples to determine the concentration of endogenous oestradiol and progesterone) to confirm the hormonal milieu in OCP users, and naturally menstruating women, a tenet that is also supported by Janse de Jonge [83]. Such measures would permit the relationship between specific ovarian hormonal profiles and exercise performance to be established. In addition, future investigations should describe the type of OCP used to the level of detail required for categorisation or replication, as different types of OCPs cause varying concentrations of endogenous sex hormones, resulting in non-homogenous participant groups [72]. The heterogeneity, caused by the non-homogenous populations plus the considerable variation in outcomes measured, likely contributed to the relatively large between study variance observed. In the future, it would be interesting to tease out which factors might cause some women to have a negative effect, while others do not, but this was not possible with the current evidence base. Future studies need to include homogenous populations, improve methodological quality and limit confounders to facilitate a deeper understanding of individual effects.

## Conclusion

Collectively, our results indicate that OCP use might result in slightly inferior exercise performance on average when compared to non-use, although any group level effect is likely to be trivial. Although most of the data used in this meta-analysis were rated as moderate to low quality (83% of the total studies), a sensitivity analysis of moderate and high quality papers (67% of the total studies) did not change the general findings described herein, thus bolstering the confidence in the evidence. From a practical perspective, as the effects tended to be trivial and variable across studies, there appears to be no performance related evidence to warrant general guidance on OCP use compared with non-use. As such, an individualised approach should be taken, based on each athlete’s response to OCP use, along with other factors such as their primary objective for using OCPs, and their experience of the naturally occurring menstrual cycle. Moreover, the difference in exercise performance between the OCP consumption and withdrawal phases was estimated on average to be close to zero, suggesting that the endogenous hormonal profile is the prevailing driver of performance rather than the supplementation of exogenous hormones. From a practical perspective, there appears to be no performance related evidence to warrant general guidance on OCP consumption versus OCP withdrawal.

## Electronic supplementary material

Below is the link to the electronic supplementary material.

Supplementary file1 (DOCX 21 kb)

Supplementary file2 (DOCX 15 kb)

Supplementary file3 (DOCX 16 kb)

## Data Availability

Please contact the corresponding author for data requests.
