# Supplementary material for: The Effects of Oral Contraceptives on Exercise Performance in Women: A Systematic Review and Meta-analysis
Source: Sports Med. 2020 Jul 14;50(10):1785–812. doi: 10.1007/s40279-020-01317-5 (PMC7497464; doi:10.1007/s40279-020-01317-5)
Supplement: Supplementary file 2 — Supplementary file2 (DOCX 15 kb) [file 40279_2020_1317_MOESM2_ESM.docx]

The Effects of Oral Contraceptives on Exercise Performance in Women: A Systematic Review and Meta-Analysis. Sports Medicine. Corresponding author: Dr Kirsty Elliott-Sale, Sport Health and Performance Enhancement (SHAPE) Research Centre, Department of Sport Science, Nottingham Trent University, Nottingham, UK. Email: [kirsty.elliottsale@ntu.ac.uk](mailto:kirsty.elliottsale@ntu.ac.uk).

| **Electronic Supplementary Material Appendix S2.** First search conducted on 09/01/19 using Pubmed. |
| --- |

| Limits applied |
| --- |
| Humans |
| Randomised controlled trials |
| Observational |
| Clinical trials |
| Clinical controlled trials |
| English language |
| Females |

| Search terms |
| --- |
| Oral contraceptives and athletic performance (19) |
| Oral contraceptives and SPORTS performance (20) |
| Oral contraceptives and muscle (38) |
| Oral contraceptives and strength (22) |
| Oral contraceptives and force (69) |
| Oral contraceptives and skeletal muscle (12) |
| Oral contraceptives and muscular strength (0) |
| Oral contraceptives and muscular force (0) |
| Oral contraceptives and power (47) |
| Oral contraceptives and anaerobic (2) |
| Oral contraceptives and anaerobic power (0) |
| Oral contraceptives and anaerobic performance (1) |
| Oral contraceptives and anaerobic capacity (1) |
| Oral contraceptives and aerobic (8) |
| Oral contraceptives and endurance (11) |
| Oral contraceptives and endurance capacity (3) |
| Oral contraceptives and endurance power (1) |
| Oral contraceptives and aerobic capacity (2) |
| Oral contraceptives and aerobic power (2) |
| Oral contraceptives and aerobic performance (1) |
| Oral contraceptives and endurance performance (4) |
| Oral contraceptives and fatigue (42) |
| Oral contraceptives and recovery (31) |
| Total = 336 with duplicates |
