# Supplementary material for: The Effects of Oral Contraceptives on Exercise Performance in Women: A Systematic Review and Meta-analysis
Source: Sports Med. 2020 Jul 14;50(10):1785–812. doi: 10.1007/s40279-020-01317-5 (PMC7497464; doi:10.1007/s40279-020-01317-5)
Supplement: Supplementary file 3 — Supplementary file3 (DOCX 16 kb) [file 40279_2020_1317_MOESM3_ESM.docx]

The Effects of Oral Contraceptives on Exercise Performance in Women: A Systematic Review and Meta-Analysis. Sports Medicine. Corresponding author: Dr Kirsty Elliott-Sale, Sport Health and Performance Enhancement (SHAPE) Research Centre, Department of Sport Science, Nottingham Trent University, Nottingham, UK. Email: [kirsty.elliottsale@ntu.ac.uk](mailto:kirsty.elliottsale@ntu.ac.uk).

| **Electronic Supplementary Material Appendix S3.** Quality Appraisal – modified Downs and Black checklist. |
| --- |

Q1. Is the hypothesis/aim/objective of the study clearly described? (Yes = 1; No = 0)

Q2. Are the main outcomes to be measured clearly described in the introduction or methods section? If the main outcomes are first mentioned in the results section, answer no. (Yes = 1; No = 0).

Q3. Are the characteristics (age, height, weight, training status, healthy) of the participants included in the study clearly described? In observational studies, inclusion and/or exclusion criteria should be given. In case-control studies, inclusion and/or exclusion and the source of controls should be given. (Yes = 1; No = 0).

Q4. Were the tested OC phase (and menstrual cycle phase if relevant) clearly described? Answer yes if the precise criteria used to define phase were provided, answer no if the exact phase tested cannot be ascertained. (Yes = 1; No = 0).

Q5. Are the main findings of the study clearly described? Simple outcome data should be reported for all major findings so the reader can check the major analyses and conclusions. This does not cover statistical tests which are addressed in other questions. (Yes = 1; No = 0).

Q6. Does the study provide estimates of the random variability in the data for the main outcomes? In non-normal data, inter-quartile range should be reported. In normal data, standard deviation, standard error or confidence intervals should be reported. (Yes = 1; No = 0).

Q7. Were the participants confirmed to be habitual pill users, or in the case of eumennorheic controls, habitual non-users, for at least 3 months prior to the study? (Yes = 1; No = 0).

Q8. Was at least one familiarization trial conducted prior to exercise testing? (Yes = 1; No = 0; Unable to determine = 0).

Q9. Were the exercise test conditions adequately standardised (factors including time of day; prior nutritional intake (including caffeine) and prior exercise)? (Yes (all relevant factors standardised) = 2; Yes (some relevant factors standardised) = 1; No = 0; Unable to determine = 0).

Q10. If any of the results of the study were based on ‘data dredging’ was this made clear? Any analyses that had not been planned at the outset should be clearly indicated. If no retrospective subgroup analyses were reported, then answer yes. (Yes = 1; No = 0; Unable to determine = 0).

Q11. Were statistical tests used to assess the main outcomes appropriate? The statistical techniques used must be appropriate to the data and the research question. (Yes = 1; No = 0; Unable to determine = 0).

Q12. Were the main outcome measures used accurate (valid and reproducible)? Answer yes for tests that have been externally validated (Yes = 1; No = 0; Unable to determine = 0).

Q13. Was the order of phase testing randomised or counterbalanced? (Yes = 1; No = 0; Unable to determine = 0).

Q15. Did the study have sufficient power to detect an a priori specified scientifically important effect at a pre-determined probability threshold? Answer yes if they included a power calculation, and no if not. (Yes = 1; No = 0).

Q16. Was study retention > 85%? (Yes = 1; No = 0; Unable to determine = 0).

The combined score was used to categorise each study outcome according to 4 categories, i.e., High (14 – 16), Moderate (10 – 13), Low (6 – 9) or Very Low (< 6)

Note: For single-measure observational trials (e.g., those that compared OC and eumenorrheic women at a single phase) questions 13 and 16 were deemed irrelevant and so were removed. The maximum attainable score for these studies was 14 and the categories were: High (12 – 14); Moderate (8 – 11); Low (4 – 7); Very Low (< 4).
